# P-474. Epidemiology of Invasive Fungal Disease in a Multicenter Pediatric Acute Myeloid Leukemia Cohort

**DOI:** 10.1093/ofid/ofaf695.689

**Published:** 2026-01-11

**Authors:** William R Otto, Anjali Khanna, Katherine Lee, Caitlin N Brammer, Caroline Maguire, Brian T Fisher, Tamara P Miller

**Affiliations:** Cincinnati Children's Hospital Medical Center, Cincinnati, OH; Emory School of Medicine, Decatur, Georgia; Children's Healthcare of Atlanta, Atlanta, Georgia; Cincinnati Children's Hospital Medical Center, Cincinnati, OH; Cincinnati Children's Hospital Medical Center, Cincinnati, OH; Children’s Hospital of Philadelphia, Philadelphia, Pennsylvania; Emory University/Children's Healthcare of Atlanta, Atlanta, Georgia

## Abstract

**Background:**

Pediatric acute myeloid leukemia (AML) patients are at risk for invasive fungal disease (IFD). Recent data on IFD for AML are from clinical trials but these data may underestimate IFD rates. Antifungal prophylaxis has become increasingly utilized during periods of prolonged, profound neutropenia, but does not prevent all IFD. Understanding the evolving epidemiology of IFD alongside increased prophylaxis use can inform need for future investigation of mitigation strategies. This study aimed to describe the real-world incidence of IFD in a contemporary multicenter AML cohort.

**Methods:**

This retrospective multicenter study included patients with AML treated at Cincinnati Children’s Hospital Medical Center, Children’s Hospital of Philadelphia, and Children’s Healthcare of Atlanta from 2011-2022. Demographic, clinical, and microbiological data were abstracted, including receipt of antifungal prophylaxis and positive mycological testing. Cases of IFD were defined using the 2020 revised EORTC/MSG consensus definitions. Incidence of proven and probable IFD was calculated for the entire cohort.

**Results:**

353 children with AML were observed for 1150 chemotherapy courses. Proven or probable IFD was identified in 8/353 (2.3%, 95% CI 1.0-4.0%) patients. IFD events included candidemia (5), disseminated *Trichosporon* infection (1), cutaneous aspergillosis (1), and pulmonary histoplasmosis (1). Patients received antifungal prophylaxis in most courses (1118/1150, 97.2%), including voriconazole (548/1118 courses, 49.0%), echinocandins (427/1118 courses, 38.2%), and fluconazole (230/1118 courses, 20.6%). Abnormal chest imaging denoting possible pulmonary IFD was common: nodules (236/648 scans, 36.4%), ground glass opacities (212/648 scans, 32.7%), consolidations (166/648 scans, 25.2%), or multifocal infiltrates (109/648 scans,16.8%).

**Conclusion:**

The rate of proven/probable IFD was low in this AML cohort that received frequent antifungal prophylaxis use during periods of neutropenia. This study provides reassuring data for IFD risk in contemporary management of AML and yields estimates of IFD rates to inform future clinical trials.

**Disclosures:**

Brian T. Fisher, DO, MPH/MSCE, Merck: Grant/Research Support|Pfizer: Grant/Research Support

